# Photosensing PUF from an Intrinsically Random SnTe Memristor for Image Encryption and Recognition

**DOI:** 10.3390/nano16120715

**Published:** 2026-06-10

**Authors:** Wendi Xu, Jia Zhang, Junjie Xie, Tianzhu Xu, Jia Wu, Hong Wang

**Affiliations:** School of Electronic Information Engineering, Hebei University, Baoding 071002, China

**Keywords:** memristor, physical unclonable function, encryption, neuromorphic computing, hardware security

## Abstract

Physical unclonable function (PUF) based on intrinsic device randomness has emerged as promising hardware security primitives, yet combining secure encryption with neuromorphic recognition within a single device platform remains challenging. Here, we demonstrate a photosensing PUF based on an intrinsically random SnTe memristor capable of both image encryption and memristive neural network recognition. The SnTe memristor, fabricated with an In_2_O_3_:SnO_2_/SnTe/Nb:SrTiO_3_ structure, exhibits stable resistive switching and stable retention exceeding 4000 s. Synaptic biomimetic behaviors including learning-experience emulation, short-term plasticity and long-term plasticity are also realized. Notably, the device displays pronounced optical sensitivity that produces stochastic photocurrent fluctuations originating from unavoidable device-to-device variations under illumination. By quantizing these random photocurrents, an encryption key stream is generated and utilized for image scrambling and diffusion. A memristive neural network is constructed to classify the encrypted images, achieving a recognition accuracy of 95.1% with a loss of 0.15 after 300 training epochs. This work establishes a viable pathway from intrinsic optical randomness to secure neuromorphic computing, highlighting the multifunctional potential of SnTe memristors in integrated hardware security and brain-inspired computation.

## 1. Introduction

The rapid advancement of information technology has placed increasing demands on both data security and computational efficiency [1,2]. Physical unclonable function (PUF) has attracted considerable attention as a hardware security primitive that exploits intrinsic physical randomness to generate device-specific cryptographic keys, offering resistance against physical attacks that threaten conventional software-based encryption [3,4,5]. In parallel, neuromorphic computing based on memristive devices has emerged as a transformative paradigm that emulates biological synaptic functions to perform energy-efficient, massively parallel computation, fundamentally addressing the von Neumann bottleneck inherent in traditional architectures [6,7,8]. Although both technologies have progressed independently, they remain largely disconnected in current practice. For emerging distributed intelligent systems where sensitive data must be securely sensed, transmitted, and recognized at the edge, an integrated platform that unifies on-chip security and energy-efficient computation is highly desirable.

Traditionally, PUF and neuromorphic computing are realized on separate platforms using distinct device technologies, making it difficult to reconcile them within a single hardware framework. Memristors, with their non-volatile resistance switching, analog conductance tunability, and nanoscale footprint, stand at the intersection of these two domains. On the one hand, the inherent cycle-to-cycle and device-to-device variability of memristors, traditionally viewed as a drawback, can be harnessed as a source of entropy for PUF implementations [9,10]. On the other hand, memristor crossbar arrays naturally perform vector-matrix multiplication via Ohm’s law and Kirchhoff’s current law, enabling direct in-memory analog computation for neural network acceleration [11,12]. Thus, memristors uniquely combine these two capabilities in one physical device, offering a rare opportunity to integrate hardware security and neuromorphic computation on the same platform.

An emerging strategy to further bridge this gap is to exploit the photoresponse of optoelectronic memristors, where light illumination introduces an additional degree of freedom for both conductance modulation and entropy generation [13,14]. Realizing this strategy, however, requires a material platform that simultaneously delivers stable electrical switching, pronounced optical response, and intrinsic randomness suitable for entropy extraction. Unlike conventional optoelectronic materials such as IGZO, MoS_2_, and ZnO, which suffer from weak visible response, high device variability, or poor stability [15,16,17], SnTe, as a narrow-bandgap IV-VI semiconductor with high carrier mobility and strong light-matter interaction, has shown promise for optoelectronic memristive applications [18,19]. Its narrow bandgap enables efficient absorption across the visible spectrum, generating substantial photocurrent even under low-intensity illumination. The high carrier mobility ensures rapid transport of photogenerated carriers to the electrodes, enhancing the overall photoresponse. These combined attributes make SnTe a compelling candidate for optoelectronic memristors aimed at unifying hardware security and neuromorphic computation.

In this work, we present a photosensing PUF based on an intrinsically random SnTe memristor integrated with a memristive neural network for image encryption and recognition. The SnTe memristor with an In_2_O_3_:SnO_2_ (ITO)/SnTe/Nb:SrTiO_3_ (NSTO) architecture exhibits stable resistive switching, and pronounced optical sensitivity. The stochastic photocurrent generated under illumination is quantified to produce an encryption key stream, which is subsequently employed for image scrambling and diffusion. The encrypted images are then classified by an artificial neural network simulated using a memristor crossbar array, achieving excellent recognition accuracy. The integrated demonstration establishes a unified platform that bridges hardware security and neuromorphic computing through intrinsic device randomness.

## 2. Materials and Methods

### 2.1. Fabrication of the SnTe Memristor

The devices in this study were fabricated on Nb-doped SrTiO_3_ single-crystal conductive substrates (Hefei Kejing Materials Technology, Hefei, China), which served as both the epitaxial growth substrate and the bottom electrode. Pulsed laser deposition (PLD, SKY Technology Development, Shenyang, China) and magnetron sputtering (Shenyang Tuopa Technology, Shenyang, China) were employed for device fabrication. Specifically, the NSTO substrates were sequentially ultrasonically cleaned in acetone (Chengdu Kelong Chemical, Chengdu, China), ethanol (Tianjin Huihang Chemical Technology, Tianjin, China), and deionized (Shanghai Aladdin Biochemical Technology, Shanghai, China) water for 5 min each to remove organic residues and particulate contaminants from the substrate surface. Subsequently, SnTe films with a thickness of approximately 17 nm were epitaxially grown on the cleaned NSTO substrates by PLD. During film deposition, a XeCl excimer pulsed laser with a wavelength of 308 nm was used at a repetition rate of 2 Hz, and the laser energy density was set to 1.5 J cm^−2^. Finally, ITO (90:10 wt%) top electrodes were deposited onto the SnTe films through a metal shadow mask by magnetron sputtering, thereby completing the fabrication of the memristor.

### 2.2. Characterization and Measurements of the SnTe Memristor

The surface morphology and homogeneity of the films were observed using scanning probe microscopy (Asylum Research MFP-3D, Asylum Research, Santa Barbara, CA, USA). The cross-section of the device was characterized by transmission electron microscope (TEM, Thermo Fisher Scientific, Waltham, MA, USA). X-ray diffraction (XRD, TD-3500, Dandong Tongda Technology, Dandong, China) was employed to characterize the phase composition, crystal structure, and preferred orientation of the device films. Electrical and optoelectronic measurements were subsequently conducted at room temperature using a semiconductor parameter analyzer (Keithley 4200, Keithley Instruments, Cleveland, OH, USA), a function generator (DG5072, RIGOL Technologies, Beijing, China), and an oscilloscope (DS4034, RIGOL Technologies, Beijing, China).

## 3. Results and Discussion

**Figure 1:**
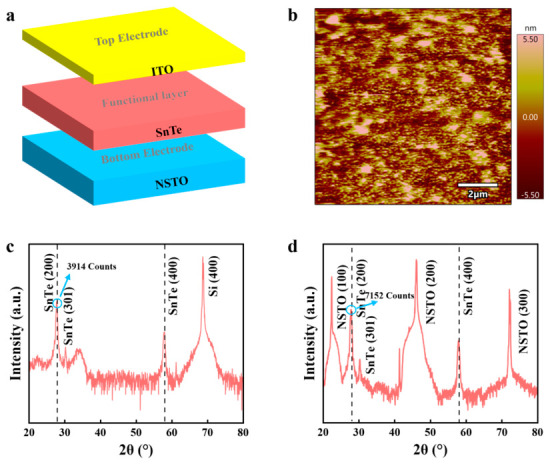
Structural and characterizations of the SnTe memristor. (**a**) Schematic diagram of the SnTe memristor. (**b**) AFM topography image of the uniform SnTe film surface. (**c**,**d**) Comparison of XRD of SnTe grown on Si and NSTO substrates.

To realize this integrated hardware platform, the SnTe memristor was first fabricated and its structural properties were systematically characterized. Figure 1a presents the schematic of the SnTe memristor, which adopts a vertically stacked configuration consisting of a conductive NSTO single-crystal substrate as the bottom electrode, a SnTe functional layer as the switching medium, and an ITO top electrode. Because surface flatness and crystalline quality critically affect memristive performance, the SnTe film was characterized by atomic force microscopy (AFM) and X-ray diffraction (XRD). The AFM topography in Figure 1b reveals a uniform and smooth film surface. This atomically smooth surface facilitates a uniform electric field distribution across the memristor active area, which is essential for achieving stable and reproducible resistive switching behavior with low cycle-to-cycle variability. Figure 1c,d compare the XRD patterns of SnTe films deposited on Si and NSTO substrates, respectively. Both patterns display diffraction peaks at 27.85°, 30.34° and 58.00°, which correspond to the (200), (301) and (400) planes of SnTe, respectively. For the film on Si (Figure 1c), the broad feature appearing between ~33° and ~35° is attributed to background scattering from the silicon substrate, and a peak at ~68.9° corresponds to the Si (400) reflection. In the pattern of the film grown on NSTO, additional peaks are observed at 22.45°, 46.15°, and 72.25°, which are indexed to the (100), (200), and (300) planes of the NSTO substrate, in agreement with previous reports [20,21]. Besides these substrate peaks, a peak at ~41.4° is indexed as a satellite peak of the NSTO substrate. Under identical growth conditions, the intensity of the SnTe (200) peak for the film on NSTO reaches 7152 counts, whereas that on Si is only 3914 counts. This marked difference indicates that the NSTO substrate promotes crystalline quality of the SnTe film. Supplementary Figure S1 shows the cross-sectional transmission electron microscope (TEM) image of the SnTe/NSTO heterostructure. The SnTe layer exhibits a uniform thickness of approximately 17 nm. The interfaces between the SnTe film and the NSTO substrate are relatively smooth and continuous, indicating good epitaxial quality.

**Figure 2:**
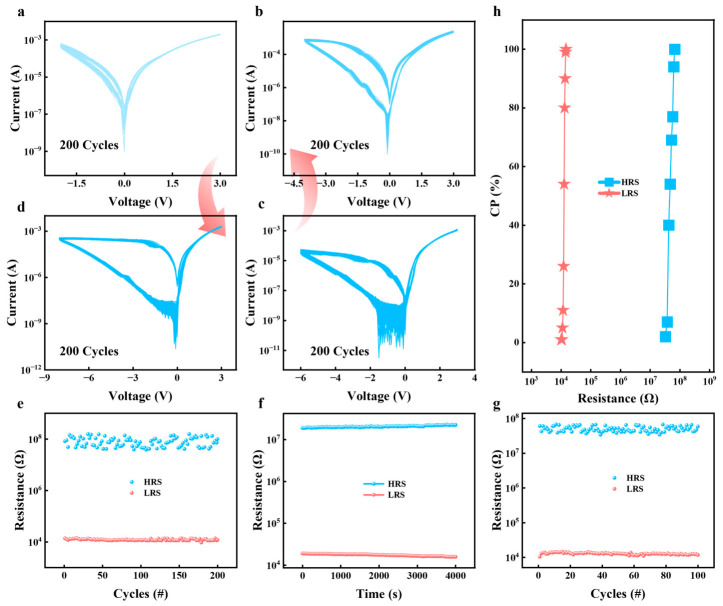
Stable resistive switching characteristics of the SnTe memristor. (**a**–**d**) *I*–*V* curves under fixed forward bias and increasing reverse bias (−2 to −8 V), with switching ratio rising at larger reverse voltages. (**e**) Resistance values of HRS and LRS extracted from 200 consecutive *I*–*V* cycles. (**f**) Stable retentions of the memristor. (**g**) Endurance properties of the device. (**h**) Cumulative probability distribution of HRS and LRS.

Having characterized the crystalline structure and smooth surface of the SnTe film grown on the NSTO substrate, the resistive switching performance of the resulting memristor was next evaluated. The NSTO electrode forms a Schottky-like contact with the SnTe layer. Under an applied voltage, the migration of intrinsic defects and carrier trapping/detrapping near the interface alter the local trapped charge distribution, thereby modulating the effective Schottky barrier height and depletion layer width. This modulation gives rise to the switching between a high-resistance state (HRS) and a low-resistance state (LRS) [22]. To further support this mechanism, the conduction behavior of the device was analyzed using the Fowler-Nordheim (FN) tunneling model. The plot of ln(*I*/*V^2^*) versus 1/*V* exhibits a linear slope in the high-voltage region, which is characteristic of FN tunneling (Supplementary Figure S2) [23]. This linearity confirms the presence of a Schottky barrier at the SnTe/NSTO interface and demonstrates that the barrier height can be modulated by the applied electric field. Such field-assisted barrier modulation is consistent with the proposed defect migration and carrier trapping/detrapping processes that lead to HRS/LRS switching. Figure 2a–d present the *I*–*V* characteristics recorded under a fixed forward bias of 3 V while sweeping the reverse bias from −2 V to −8 V in steps of −2 for 200 consecutive cycles. The fixed forward bias is chosen to provide a consistent set process, while the reverse bias sweep is used to probe the reset behavior and assess the voltage dependence of the switching performance. It is clearly observed that the switching ratio in the negative bias region increases progressively with increasing reverse voltage, and the device’s curve remains highly stable over all 200 cycles, with negligible drift in the overall shape. The increase in switching ratio can be understood as follows: a larger reverse bias drives more pronounced barrier modulation and more complete reset of the conductive region, yielding a higher HRS resistance while the LRS resistance remains essentially unchanged, thus resulting in an enlarged HRS/LRS ratio. Based on the *I*–*V* curves at −8 V (Figure 2d), the resistance values of the HRS and LRS were extracted from the 200 successive cycles. As shown in Figure 2e, both the HRS and LRS are tightly clustered with minimal cycle-to-cycle fluctuations. The stable behavior of the device is further demonstrated by the stable data retention over 4000 s in Figure 2f, during which both the HRS and LRS resistances show no significant degradation, indicating the non-volatility of both states. To verify the stability of the device, the endurance of the device is further studied under pulse mode stimulation (−1.8 V, 1 μs; −0.1 V, 1 μs). As shown in Figure 2g, the device can realize 100 transitions between the high and low resistance state without significant resistance decay, with the HRS/LRS ratio remaining distinguishable across all 100 cycles, indicating that the device has good stability. Moreover, the cumulative probability distributions of the HRS and LRS, extracted from a single device over repeated switching cycles, are clearly separated (Figure 2h), further confirming the stable two-state distinction required for memory and logic applications.

**Figure 3:**
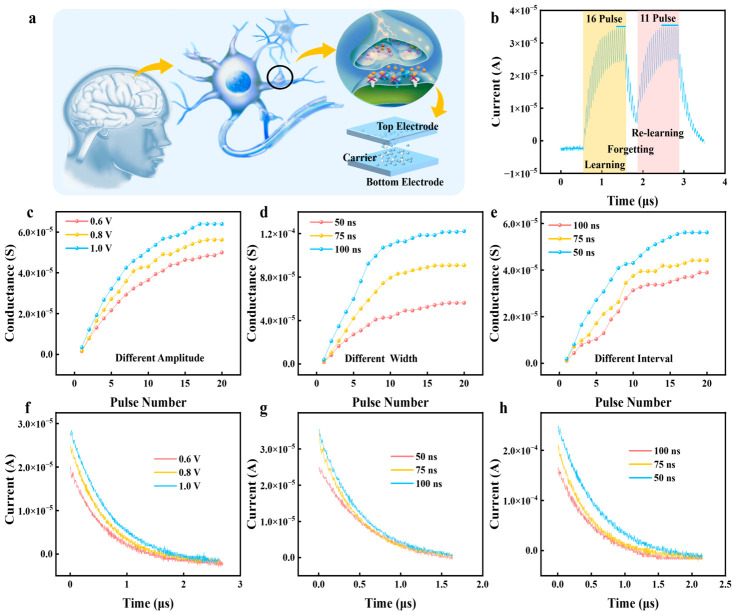
Synaptic biomimetic behavior of the SnTe memristor. (**a**) Schematic illustration of a neural synapse. (**b**) Simulation of the learning-experience process. (**c**–**e**) Conductivity regulation behaviors using electrical pulses with different amplitudes, widths, or intervals. The fixed parameters are: width and interval at 50 ns (for amplitude variation); amplitude at 0.8 V and interval at 50 ns (for width variation); amplitude at 0.8 V and width at 50 ns (for interval variation). (**f**–**h**) EPSC behaviors of electrical synapses at different conditions.

In the SnTe memristor, the electrically tunable conductance provides the essential basis for biomimetic synaptic emulation. Figure 3a shows a schematic of a biological synapse. In such a synapse, signals are transmitted across the synaptic cleft via the release and reception of neurotransmitters, a process that bridges the electrical signal of the presynaptic neuron to the postsynaptic neuron. Analogously, in the SnTe memristor, the top electrode serves as the signal-input terminal akin to the presynaptic membrane, the bottom electrode as the signal-receiving terminal akin to the postsynaptic membrane, and the SnTe functional layer acts as the transmission medium analogous to the synaptic cleft. Under external bias, the migration and redistribution of charge carriers within the SnTe layer modulate the device conductance, mimicking the neurotransmitter-mediated modulation of synaptic weight in biological systems.

Against this backdrop, Figure 3b simulates the learning-experience process. The initial learning process required 16 electrical pulses to reach a given conductance level, whereas after a brief forgetting period, only 11 pulses were needed during relearning to restore the same level. This closely mirrors the biological behavior where prior experience facilitates faster re-learning. Further demonstrating the plasticity of the artificial synapse, Figure 3c–e show that the conductivity can be continuously regulated by tuning the amplitude, width, and interval of the applied pulses. Specifically, larger pulse amplitudes produce greater conductance changes per pulse, enabling coarse adjustment of the synaptic weight. Longer pulse widths extend the effective duration of each stimulation, resulting in larger cumulative conductance modulation. Shorter pulse intervals limit the relaxation time between successive stimuli, leading to a more rapid accumulation of conductance change. The independent tunability of these three parameters allows precise control over the potentiation and depression characteristics, which is essential for implementing accurate synaptic weight updates during neural network training. To quantitatively evaluate the conductance update linearity and symmetry, we performed long-term potentiation (LTP) and long-term depression (LTD) measurements on the SnTe memristor (Supplementary Figure S3). LTP was realized by applying continuous positive voltage pulses (0.6 V, pulse width 100 ns, interval 100 ns), while LTD was induced by continuous negative voltage pulses (−1.2 V, pulse width 150 ns, interval 100 ns). The extracted linearity values are 91.9% for LTP and 97.5% for LTD, indicating good update linearity and symmetry, which are favorable for neural network training. The excitatory postsynaptic current (EPSC) responses recorded under various stimulation conditions (Figure 3f–h) also reproduce key short-term plasticity (STP) features. When two consecutive pulses are applied with a short interval, the EPSC amplitude evoked by the second pulse is markedly larger than that of the first, demonstrating paired-pulse facilitation. As the inter-pulse interval increases, the facilitation effect gradually diminishes and eventually transitions to paired-pulse depression at longer intervals. Furthermore, the EPSC amplitude increases with increasing pulse amplitude and width, and decays back to the baseline after the stimulus is removed, with the decay time constant reflecting the intrinsic relaxation property of the device. These EPSC characteristics closely resemble the dynamic postsynaptic responses observed in biological synapses, including the dependence of facilitation strength on stimulation frequency and the transient nature of postsynaptic current. Collectively, these results validate the direct biomimetic correspondence between the memristor and biological synapses.

**Figure 4:**
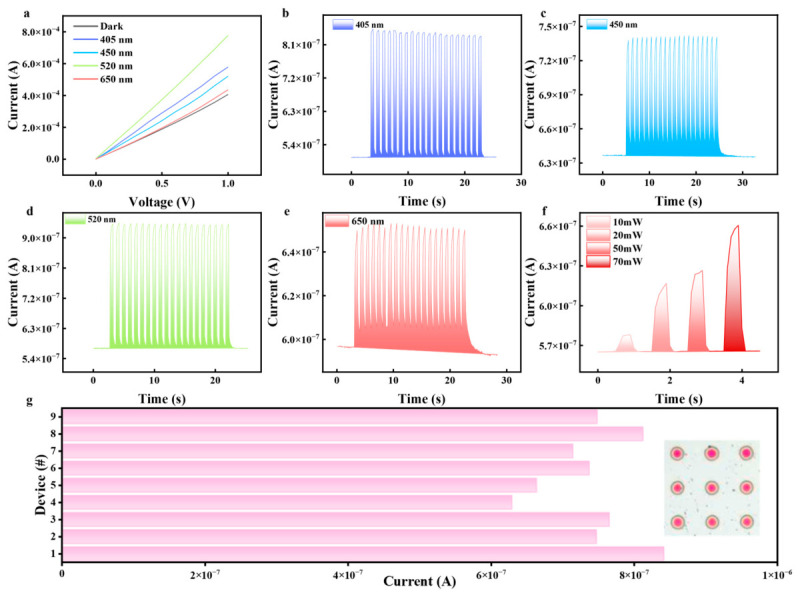
Optical properties of the SnTe memristors. (**a**) *I*–*V* diagram of unidirectional scanning under different lighting conditions. (**b**–**e**) Different optical responses under different-wavelength light signals (405/450/520/650 nm). (**f**) Power-dependent photocurrent response at wavelengths of 650 nm. (**g**) Photoresponse of a 3 × 3 memristor array under 405 nm illumination.

In addition to the synaptic biomimetic behaviors, the SnTe memristor also exhibits notable optical sensitivity, extending its functionality toward optoelectronic applications. This photoresponse originates from the semiconducting nature of SnTe, which can generate electron–hole pairs upon light absorption. Figure 4a shows the *I*–*V* curves recorded under dark conditions and under illumination at different wavelengths. It is clearly observed that light irradiation increases the device current compared with the dark state, and the enhancement is strongly wavelength-dependent. The results indicate that the device exhibits its strongest response at 520 nm and its weakest at 650 nm. The stronger response at 520 nm is attributed to its higher photon energy, which enables photoexcited carriers to gain sufficient kinetic energy to overcome the Schottky-like barrier at the SnTe/NSTO interface, whereas the lower photon energy at 650 nm results in a smaller fraction of carriers with enough energy to contribute to the photocurrent [24]. Subsequently, the output signal responses of the device under different light signals were quantitatively compared, as shown in Figure 4b–e. Furthermore, the device exhibits power-dependent behaviour, with the device current showing a monotonically increasing trend as the incident light power increases (Figure 4f). In addition to these steady-state photoresponse characteristics, the photocurrent measured under constant illumination exhibits stochastic device-to-device fluctuations arising from the inherent variations among individual devices, which provide a suitable entropy source for PUF, as will be exploited in the following encryption demonstration. Figure 4g shows the photocurrent response of a 3 × 3 memristor array under 405 nm illumination.

The coexistence of pronounced optical sensitivity and synaptic biomimetic behaviors in a single SnTe memristor presents an opportunity to merge hardware security with neuromorphic computing on a unified platform. Conventional implementations typically deploy discrete components for cryptographic key generation and for neural network computation, which not only increases system complexity but also introduces potential vulnerabilities at the interfaces between separate modules. By contrast, an optoelectronic memristor that simultaneously exhibits stochastic photocurrent fluctuations under illumination and analog conductance tunability under electrical operation can serve both as an entropy source for physical unclonable functions and as a synaptic element for in-memory computing. This dual functionality allows the same device array to perform encryption key extraction and neural network inference, eliminating the need for separate security and processing hardware. The following demonstration illustrates this integrated concept by first constructing a photosensing PUF for image encryption and subsequently implementing a memristive neural network for classifying the encrypted images.

**Figure 5:**
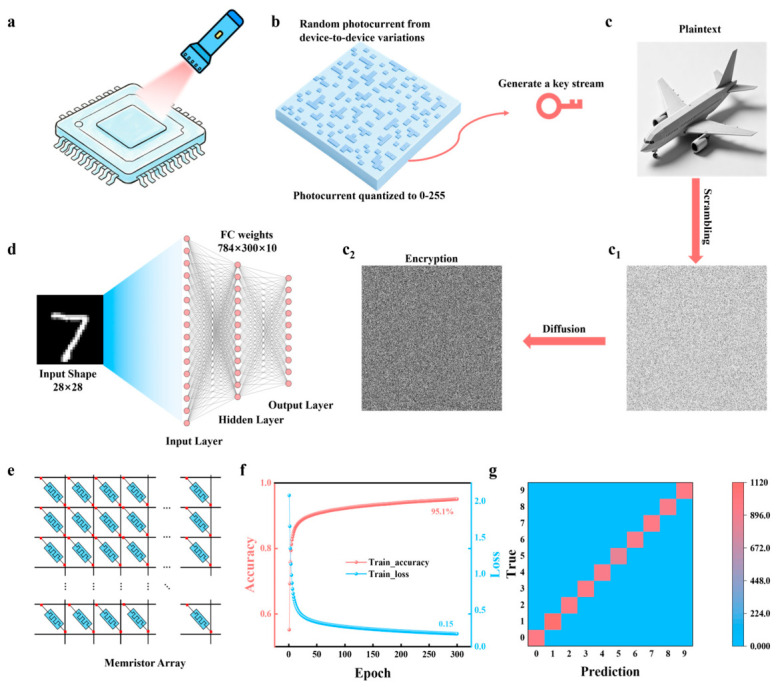
Encryption via SnTe photosensing memristor PUF and recognition by memristive neural network. (**a**,**b**) Key stream generation using the photosensing PUF based on an intrinsically random SnTe memristor. (**c**) Encrypted image after scrambling and diffusion, where c_1_ is the image after scrambling and c_2_ is the image after diffusion. (**d**) Schematic of the artificial neural network architecture. (**e**) Schematic diagram of the memristor array. (**f**) Training accuracy curves of the memristive neural network. (**g**) Confusion matrix for classification results.

To implement this integrated concept, a photosensing memristor PUF is constructed for image encryption. Figure 5a,b present an encryption scheme based on a photosensing memristor PUF. Under constant illumination, different SnTe memristors fabricated under identical process conditions generate photocurrents that exhibit stochastic fluctuations originating from unavoidable device-to-device variations, rather than from instability of a single device. These random current signals are sampled and quantized into integer values ranging from 0 to 255, forming an encryption key stream with a bit depth matching that of the original image pixels. Figure 5c illustrates the encryption process, in which the original image is first scrambled by permuting the pixel positions according to the key stream, and then diffused via a bitwise exclusive OR (XOR) operation between the scrambled pixel values and the key stream, yielding the final encrypted image that is uncorrelated with the original. As a synaptic biomimetic device, the memristor plays a pivotal role in neuromorphic computing systems. Figure 5d presents a schematic of the artificial neural network architecture. The network consists of an input layer, multiple fully connected hidden layers, and an output layer for classification. Specifically, the input image of 28 × 28 pixels is flattened into a 784-dimensional vector, which is fed into a fully connected hidden layer with 300 neurons, followed by an output layer of 10 neurons corresponding to the classification categories. Neural network training and inference inherently involve massive numbers of multiply–accumulate (MAC) operations. Notably, memristor crossbar arrays offer a native physical realization of these operations. By mapping the synaptic weight matrix onto the conductance values of the crossbar array, the input vector, encoded as voltage pulses applied to the word lines, is multiplied by the corresponding conductance at each crosspoint according to Ohm’s law, yielding current outputs proportional to the product. These output currents then naturally accumulate along the bit lines following Kirchhoff’s current law, thereby completing a full vector-matrix multiplication (VMM) operation in a single step. This physical VMM is intrinsically parallel, as all crosspoint multiplications and column-wise summations occur simultaneously across the entire array, independent of the matrix dimensions. This in-memory analog computation fundamentally eliminates the data shuttling bottleneck inherent to conventional von Neumann architectures, enabling massively parallel and energy-efficient synaptic computation [25]. Leveraging this inherent physical advantage, the memristor array in our work (Figure 5e) was configured in simulation to implement the synaptic layers of the neural network, allowing all weight storage and MAC operations to be performed directly within the array itself. Figure 5f presents the training and recognition performance of the memristive neural network. After 300 training epochs, the network achieves an accuracy of 95.1% with a loss of 0.15, as shown in the training curves, while the confusion matrix (Figure 5g) further confirms the reliability of the encrypted image classification, with high diagonal values demonstrating correct classification across most categories. These results establish a unified hardware platform that bridges intrinsic optical randomness and memristive neural recognition, opening a viable route toward integrated secure neuromorphic computing.

## 4. Conclusions

In summary, a SnTe memristor that integrates hardware security and neuromorphic computing on a unified platform has been demonstrated. The device exhibits stable resistive switching with a clear HRS/LRS distinction over 200 consecutive cycles, retention exceeding 4000 s, and reliable endurance over 100 pulse-driven transitions. For neuromorphic functionality, synaptic biomimetic behaviors including learning-experience emulation, pulse-parameter-dependent conductance modulation, and short-term plasticity are realized under electrical operation, confirming that the device can serve as a synaptic element with analog weight tunability. For hardware security, pronounced optical sensitivity is harnessed: under constant illumination, the device generates stochastic photocurrent fluctuations, which are quantized into an encryption key stream for image scrambling and bitwise XOR diffusion. The encrypted images are subsequently classified by a memristor crossbar-based neural network directly, achieving a recognition accuracy of 95.1% with a loss of 0.15 after 300 training epochs. Notably, the same memristor device simultaneously provides the entropy source for key generation and the synaptic element for neural computation, thereby unifying data encryption and neuromorphic recognition within a single hardware platform. This work establishes a continuous pipeline from intrinsic optical randomness to secure in-memory neuromorphic recognition and demonstrates that the convergence of optoelectronic sensing, hardware security, and brain-inspired computing within a single memristive device is feasible. Such a unified architecture points toward a new paradigm for distributed intelligent edge systems, where sensing, encryption, and computation are natively fused at the hardware level, eliminating the traditional boundaries between secure data acquisition and energy-efficient processing.

## Data Availability

The original contributions presented in this study are included in the article/Supplementary Material. Further inquiries can be directed to the corresponding author.

## Supplementary Materials

The following supporting information can be downloaded at: https://www.mdpi.com/article/10.3390/nano16120715/s1, Figure S1: TEM cross-section diagrams of ITO/SnTe/NSTO memristor; Figure S2: Mechanism fitting: FN tunneling plot with R^2^ = 96.3; Figure S3: Realization of LTP and LTD synaptic characteristics under continuous positive pulse stimulation (0.6 V, 100 ns, 100 ns) and negative pulse stimulation (−1.2 V, 150 ns, 100 ns).
