# Supplementary material for: Photosensing PUF from an Intrinsically Random SnTe Memristor for Image Encryption and Recognition
Source: Nanomaterials (Basel). 2026 Jun 10;16(12):715. doi: 10.3390/nano16120715 (PMC13305159; doi:10.3390/nano16120715)
Supplement: Supplementary file 1 [file nanomaterials-16-00715-s001.zip › nanomaterials-4340577-supplementary.pdf]

Supporting Information

Photosensing PUF from an Intrinsically Random SnTe Memristor for Image Encryption and Recognition

Wendi Xu[1], Jia Zhang[1], Junjie Xie[1], Tianzhu Xu[1], Jia Wu[1,*], Hong Wang[1,*]

[1]*School of Electronic Information Engineering, Hebei University, Baoding 071002, China.*

1. Supplementary Figure S1-3

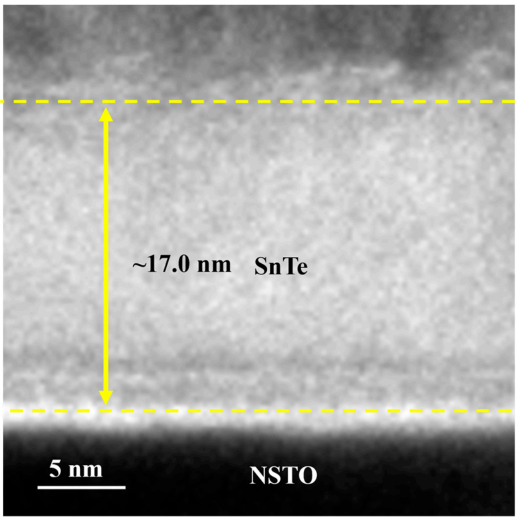

Supplementary Figure S1. TEM cross-section diagrams of ITO/SnTe/NSTO memristor.

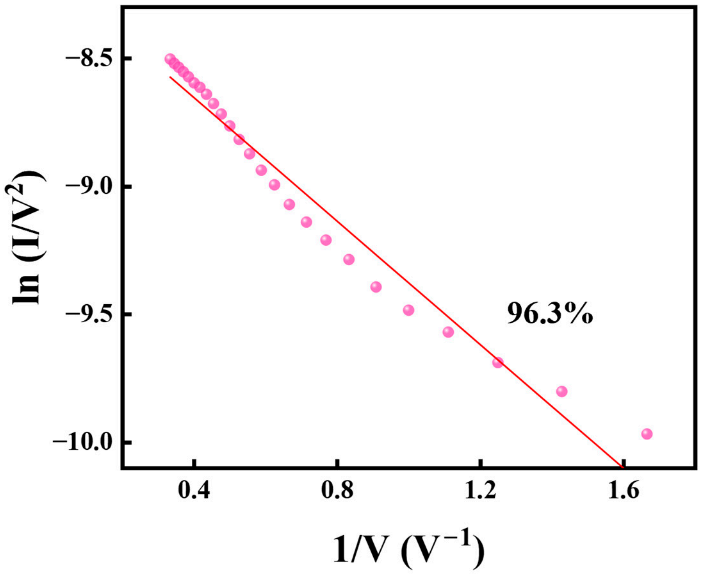

Supplementary Figure S2. Mechanism fitting: FN tunneling plot with R^2=96.3.

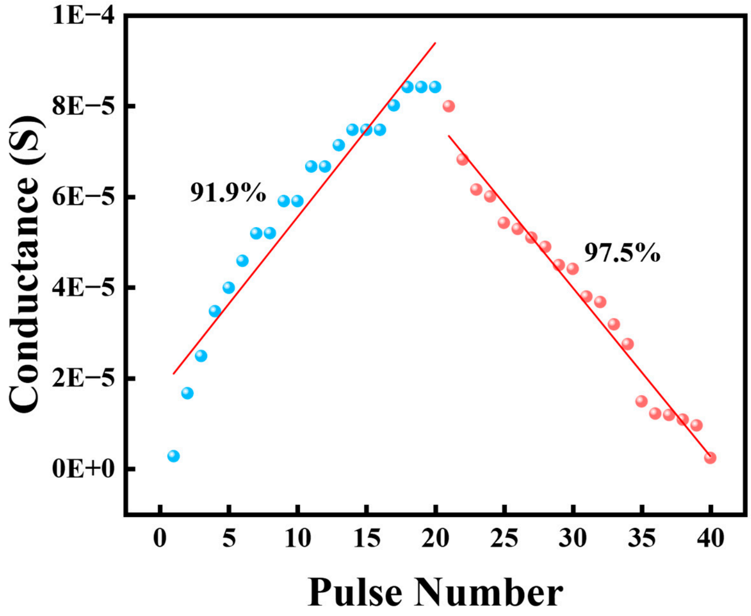

Supplementary Figure S3. Realization of LTP and LTD synaptic characteristics under continuous positive pulse stimulation (0.6 V, 100 ns, 100 ns) and negative pulse stimulation (−1.2 V, 150 ns, 100 ns).
